# Spatio-temporal analysis of genetic diversity in the sibling species *Contracaecum osculatum* sp. B: a tool for monitoring trophic-web dynamics in Arctic Sea waters

**DOI:** 10.1007/s00436-025-08561-6

**Published:** 2025-11-29

**Authors:** Beatrice Belli, Marialetizia Palomba, Veronica Fernandez-Rodriguez, Paolo Cipriani, Lucilla Giulietti, Miguel Bao, Giuseppe Nascetti, Simonetta Mattiucci

**Affiliations:** 1 Department of Public Health and Infectious Diseases, Sapienza University of Rome, Rome, Italy; 2 Department of Ecological and Biological Sciences, Tuscia University Viterbo Italy, Viterbo, Italy; 3 Institute of Marine Research (IMR) Bergen Norway , Bergen, Norway

**Keywords:** *Contracaecum osculatum* sp. B, Fish, Seals, Microsatellite DNA loci, mtDNA *cox*2, Genetic structure, Genetic variability, Trophic-web

## Abstract

Global changes in the marine Arctic ecosystem affecting the demography of fish and seal populations may also alter their anisakid parasite populations size, potentially leading to genetic erosion in gene pools. *Contracaecum osculatum* sp. B, an Arctic member of the *C. osculatum* (s.l.) species complex, parasitizes Arctic and sub-Arctic seals and fish. This study aimed to compare parasite genetic diversity across the Nordic Seas over time, using historical specimens (1985–1986) and contemporary ones (2021–2022) obtained from seals and fish of the same regions. Parasites were first identified by sequence analysis of mtDNA *cox2* and ITS rDNA, then genotyped at seven SSRs nuclear DNA loci, developed in the present study. The population genetic structure of *C. osculatum* sp. B revealed the existence of two parasite subpopulations, a pattern that appears to be mostly influenced by the population structure of the seal hosts in the area. High genetic polymorphism was observed in the parasite species, at both nuclear and mitochondrial level. In the contemporary parasite population gene pool, some rare haplotypes in mtDNA *cox*2 and rare alleles at SSRs-DNA loci appeared to be lost in comparison to the historical population; while, novel unique alleles and haplotypes simultaneously emerged. Overall, these findings would suggest the occurrence of an initial decline in parasite population size, followed by a period of demographic stability, and a population increase in recent years. Similar demographic patterns have been documented in the host populations over the same time scale. Genetic polymorphisms in anisakids across temporal scales may help unravel and monitor trophic-web dynamics in the Arctic marine ecosystem under global change.

**Supplementary Information:**

The online version contains supplementary material available at 10.1007/s00436-025-08561-6.

## Introduction

Trophically transmitted marine helminths, which have heteroxenous life cycles with free-living stages and multiple hosts across different trophic levels, are recognized as ecological indicators of food web interactions and ecosystem health (Lafferty et al. 2008a,b; Mattiucci and Nascetti 2008; Mattiucci et al. 2015; Palomba et al. 2023). Closely linked to trophic transmission and host specificity, these helminth endoparasites require stable trophic webs, as well as diverse and abundant definitive and intermediate host populations to complete their life cycle (Marcogliese 2005; Mattiucci and Nascetti 2008; Palomba et al. 2023; Wood & Vanhove 2023). Any anthropogenic stressor that causes a decline in host populations may also affect the population size of their endoparasites (Marcogliese 2005; Nichols and Gómez 2011; Palomba et al. 2023) Thus, parasite population size can be expected to fluctuate in response to global change effects (Mattiucci and Nascetti 2008; Altman and Byers 2014; Wood and Vanhove 2023).

Among these stressors, over-exploitation of natural resources, such as overfishing, may reduce host density, thereby decreasing parasite transmission and abundance (Dobson and May 1987; Lafferty et al. 2008b). In addition, both natural (e.g. virus spread) and anthropogenic impacts (e.g. hunting, by-catch, ship collisions, noise disturbance, pollution) affecting definitive hosts, such as marine mammals (e.g. cetaceans and pinnipeds), may reduce their population size (Härkönen et al. 2006; Blanchet et al. 2021; Silva et al. 2021), thereby decreasing parasite recruitment. Other anthropogenic effects, such as pollution, may weaken host immune defences against specific parasite species, leading to increased transmission, or may have the opposite effect by causing direct mortality of free-living infectious stages (Sures and Nachev 2022). Rising ocean temperatures associated with climate change may further alter host population abundance, distribution, and migratory routes, consequently affecting parasite transmission and distribution (King et al. 2015; Levsen et al. 2018, 2022; Palomba et al. 2023; Díez et al. 2024; Mattiucci et al. 2025a). Indeed, rising ocean temperature may also directly affect the free-living stages of parasites, impacting their survival, development rates, and dispersal, altering population size and distribution patterns. Overall, these phenomena impact parasite genetic polymorphism and variability (Mattiucci and Nascetti 2008; Mattiucci et al.2025a), potentially leading to a loss of genetic diversity and polymorphism in the gene pool of a heteroxenous marine parasite population due to genetic drift (Bullini et al. 1986; Mattiucci and Nascetti 2008; Mattiucci et al. 2025a).

The Arctic marine ecosystem represents a valuable model for wildlife ecology studies, allowing for comparative analysis with other oceanographic areas. Arctic parasites, like their hosts, face new challenges as climate change alters host-parasite interactions (Davidson et al. 2011). In this context, anisakid nematodes have been previously proposed as indirect indicators of trophic web stability in marine ecosystems across spatial and temporal scales, based on measurable features such as genetic variability and parasitic load estimates (Mattiucci and Nascetti 2008; Mattiucci et al. 2015; Palomba et al. 2023). However, identifying long-term trends in marine endoparasites remains challenging due to incomplete timeline data, variability in study methodologies, and inconsistencies in temporal genetic analyses.

In Arctic and sub-Arctic waters, three sibling species of the *C. osculatum* (s.l.) complex—*C. osculatum* sp. A, *C. osculatum* sp. B, and *C. osculatum* (s.s.) -occur in seals and fish species. These species are reproductively isolated and exhibit differential host preferences (Nascetti et al. 1993). The existence of these parasite species, discovered by allozyme markers (Nascetti et al. 1993), was later confirmed by sequences analysis of the ITS region of rDNA (Zhu et al. 2000) and the mitochondrial cytochrome C oxidase subunit II (mtDNA *cox2*) gene locus (Mattiucci et al. 2008). In particular, *C. osculatum* sp. B, found in the North East and North West Arctic and sub-Arctic waters, has a life cycle that primarily involves the harp seal (*Pagophilus groenlandicus*), the common seal (*Phoca vitulina*) and to a lesser extent, the grey seal (*Halichoerus grypus*) as definitive hosts (Mattiucci and Nascetti 2008; Gabel et al. 2021). Fish species, such as capelin and Arctic cod, serve as intermediate or paratenic hosts (Levsen et al. 2016, 2022; Gay et al. 2018).

In the present study, the genetic variation of the parasite *C. osculatum* sp. B was analyzed in Arctic Sea waters across seal and fish hosts and over an extended time scale in the aim to: *i)* assess the population genetic structure of *C. osculatum* sp. B using nuclear newly developed DNA microsatellite loci and mitochondrial (mtDNA *cox*2) loci, based on historical (1985–1986) and contemporary (2021–2022) samples, and *ii)* compare genetic variability estimates in the population from the two temporal periods, using both mitochondrial and nuclear genetic datasets, in order to explore variation of the parasite genetic variability that may result from changes in parasite population size driven by host demographic shifts in Arctic Sea waters.

## Materials and methods

### Nematodes sampling

**Fig. 1:**
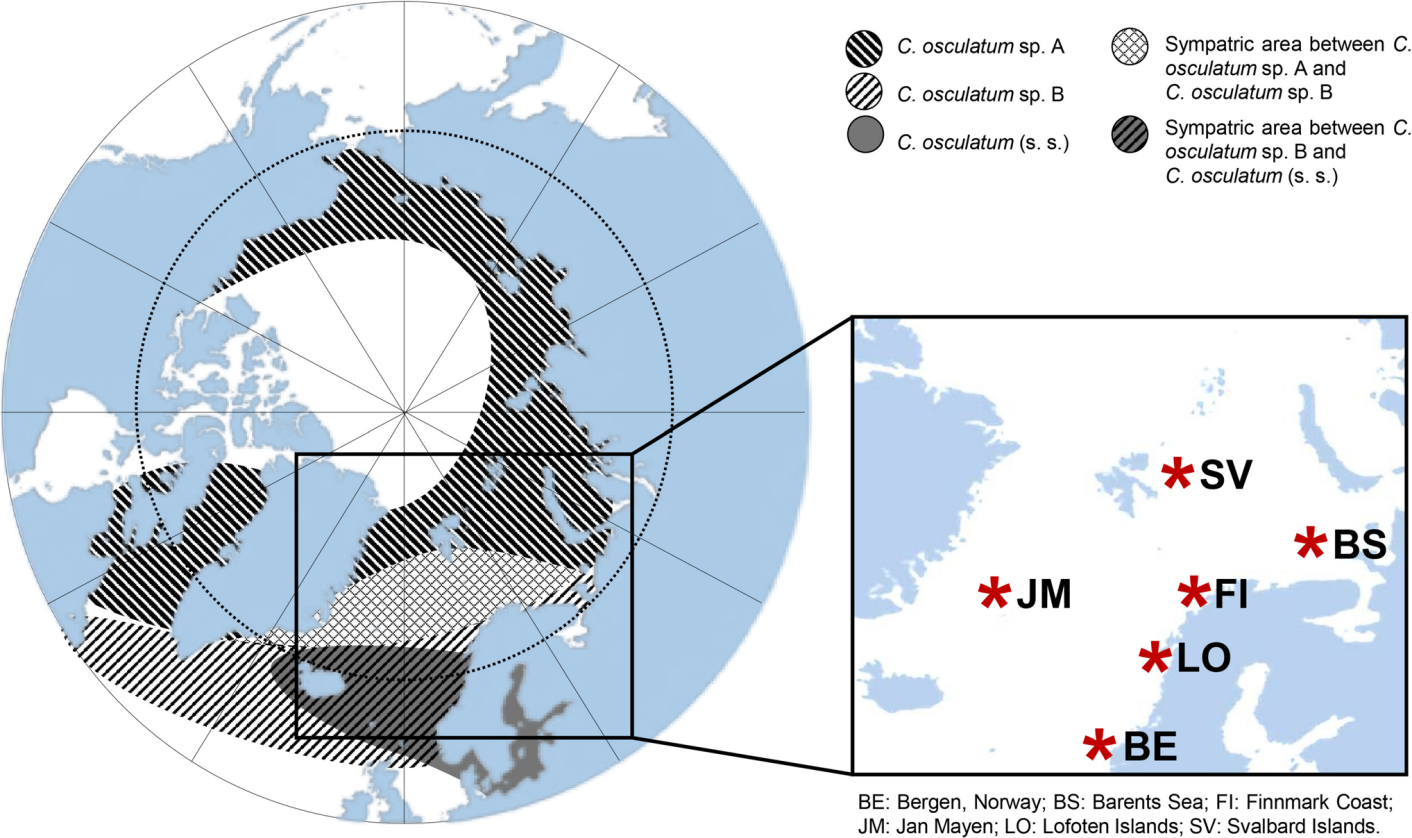
Collecting sites of specimens *C. osculatum* (s.l.) analyzed in the present study, mapped into the geographical range of the three Arctic sibling species, i.e., *C. osculatum* sp. A, *C. osculatum* sp. B and *C. osculatum* (s.s.), as reported in Nascetti et al. (1993)

A total of N = 602 anisakid specimens of *Contracaecum osculatum* (s.l.) were genetically analyzed. This included N = 388 nematodes obtained from the historical reference collection established and curated by the corresponding author at the Section of Parasitology, Department of Public Health and Infectious Diseases, Sapienza University of Rome. Adult specimens were collected from several harp seal (*Pagophilus groenlandicus*) individuals and a single harbour seal (*Phoca vitulina*) from the Nordic Seas during the years 1985–1986 (Table 1; Fig. 1). Adult *Contracaecum osculatum* specimens were first assigned as male and female based on the morphological characters described by Fagerholm (1989). The anisakid nematodes in the contemporary sample (N = 214) were collected at the L3 larval stage from N = 35 Arctic cod (*Gadus morhua*) and pollock (*Pollachius virens*) fished in Norwegian Arctic and sub-Arctic regions between 2021 and 2022 (Table 1; Fig. 1).

**Table 1:** Number of specimens of *C. osculatum* (s. l.) scored in the present study at different mitochondrial and nuclear gene loci, reported with their host species, life-history stage and sampling areas

**Sampling period**	**Area**	**Code area**	**Host species**	**N**_**H**_	**N**_**P**_	**N**_**A**_	**N**_**L3**_	**N** ***cox*2 mtDNA**	**N ITS rDNA**	**7 SSRs DNA loci**
1985-86	Barents Sea (69°22’N-39°30’E)	BS	*Pagophilus groenlandicus*	6	135	135	-	92	92	92
	Bergen (Norway)	BE	*Pagophilus groenlandicus* *Phoca vitulina*	5 1	116 16	116 16	- -	24 12	24 12	24 12
	Jan Mayen (70°44’N-7°44’W)	JM	*Pagophilus groenlandicus*	3	121	121	-	80	80	80
			Tot. (1985-86)	15	388	388	-	208	208	208
2021-22	Barents Sea (75°15’N-3°17’E)	BS	*Gadus morhua*	6	60	-	60	60	60	60
	Finnmark Coast (71°13’N-24°46’E)	FI	*Gadus morhua*	12	50	-	50	50	50	50
			*Pollachius virens*	4	10	-	10	10	10	10
	Lofoten Islands (68°64’N-14°13’E)	LO	*Gadus morhua*	9	64	-	64	64	64	64
	Svalbard Islands (72°02’N-03°01’E)	SV	*Gadus morhua*	4	30	-	30	30	30	30
			Tot. (2021-22)	35	214	-	214	214	214	214
			Tot.		602	388	214	422	422	422

NH, number of individual hosts examined; NP, total number of parasites tested; NA, number of adult nematodes; NL3, number of specimens at third larval stage

Samples underwent multiple washes in ultrapure water before DNA extraction. DNA was successfully obtained from N = 208 historical and N = 214 contemporary specimens out of the N = 602 tested. Ethanol-preserved samples were pre-treated with multiple washes in 9% Phosphate Buffered Saline (PBS) over 24–48 h. Total DNA was extracted from a tissue portion of each larval specimen. Only the internal body parts of adult worms were used for DNA extraction, while the cephalic and caudal extremities were preserved in alcohol. DNA extraction followed the protocol recommended by the Quick-gDNA Miniprep Kit (ZYMO RESEARCH).

A total of N = 422 *C. osculatum* (s.l.), including both the historical and contemporary specimens from definitive and intermediate/paratenic hosts (Table 1), were sequenced at two diagnostic genes i.e. the ITS region of rDNA (Zhu et al. 2000; Nadler et al. 2005) and the mtDNA *cox*2 gene locus (Mattiucci et al. 2008). For the ITS region of rDNA, the primers NC5 (5’ – GTA GGT GAA CCT GCG GAA GGA TCA TT – 3’) and NC2 (5’ – TTA GTT TCT TTT CCT CCG CT – 3’) were used, following the PCR condition as reported in Zhu et al. (2000). The mtDNA *cox*2 was amplified using the primers 211 F (5’ – TTT TCT AGT TAT ATA GAT TGR TTY AT—3’) and 210R (5’—CAC CAA CTC TTA AAA TTA TC – 3’) (Nadler and Hudspeth 2000), following the PCR conditions described in Mattiucci et al. (2008, 2015).

### Development of DNA microsatellites loci (SSRs-DNA)

A subset of *C. osculatum* sp. B from contemporary samples, previously identified through ITS rDNA and mtDNA *cox*2 sequence analyses, was first used to generate a highly concentrated pooled DNA sample suitable for genome library construction and scoring of the SSRs-DNA loci. A whole-genome sequencing library was created using the Illumina TruSeq Nano DNA Library Kit and sequenced on an Illumina MiSeq platform with a Nano v2 500-cycle sequencing chip. After assembly, 99,743 merged reads were screened, of which 10,490 contained microsatellite inserts with tetra- or tri-nucleotide repeats of at least six units, or dinucleotide repeats of at least ten units. From these, primer design was successfully performed for 5,870 microsatellite candidates. A total of seven microsatellite loci were then selected and named as: *Co42335*, *Co85484*, *Co97601*, *Co121208*, *Co166121*, *Co210159*, *Co215165* (Table 2). Primers were designed for an optimal annealing temperature of 56 °C and tested for polymorphism using 15 pooled *C. osculatum* sp. B specimens from various geographical areas. Allele size was determined on an ABI3730, using the GeneScan™−500 LIZ Size Standard. In the initial test for SSRs development using the subset of *C. osculatum* sp. B samples, the number of alleles ranged from 4 to 11, ensuring a good level of polymorphism in the parasite species. Subsequently, all the adult and larval *C. osculatum* sp. B specimens (reported in Table 1) previously sequenced at the mtDNA *cox*2 and ITS region of rDNA, were analyzed using the seven SSRs DNA loci (Table 2). Multiplex PCR amplification was optimized in a 10 μl reaction volume, containing 2–10 ng of genomic DNA, 5 μl Multiplex Master Mix (Qiagen®), double-distilled water, and 0.3 μM labeled forward and reverse primers (Table 2). The following cycling protocol was used for amplification: 35 cycles at 94 °C for 30 s, 56 °C for 90 s, and 72 °C for 60 s. A prolonged denaturation step (95 °C for 15 min) was included before the first cycle, and the final cycle was followed by a 30-min extension at 72 °C. Amplified PCR products were genotyped by an external company (Bio-Fab Research©). Electropherograms were manually checked using PeakScanner v.2 (Applied Biosystems, Waltham, MA, USA). The software MICRO-CHECKER v.2.2.3 (Van Oosterhout et al. 2004) was used to check for the presence of genotyping errors and null alleles.

**Table 2:** Locus name, primers sequences, number of alleles (N_A_), repeat type, fragment bp size and fluorescent dye of 7 DNA microsatellite loci (SSRs-DNA) developed in the species *C. osculatum* sp. B

**Locus name**	**Primer sequence 5’ – 3’**	**N**_**A**_	**Repeat type**	**Range size (bp)**	**Dye**
*Co42335*	F: AAGTTTAGGGCGATTGCACG* R: AAGTGATCAACGACGAAGGC	11	(GCA) 8	65–95	ATTO565 (red)
*Co215165*	F: ACCAAGAAACGCATTCGTGC* R: ACAGCCAGCATTTGGTATCG	11	(TACT) 8	110–154	ATTO532 (green)
*Co97601*	F: GAAATAAGTCCAGGTGCACGG* R: AAATTCATCCTTTTGCGTTGAC	11	(ACA) 7	107–167	FAM (blue)
*Co85484*	F: TTATAACACGCCTTCGGAGC* R: GGGGACAAAAGCGTATCGTG	24	(TAA) 11	101–203	ATTO565 (red)
*Co121208*	F: TGAAGCAGCATCTACCATTTCG* R: AATAGTAGCAGTGAAACACGTC	7	(TGT) 8	154–172	ATTO550 (yellow)
*Co210159*	F: ACTGTATAACGACCACATAGAGC* R: ACGTATTAGCGATCTTCAACAGG	24	(TTA) 7	125–272	ATTO532 (green)
*Co166121*	F: ACGTCCTCATAAATGCCCAAAC* R: GCAGTGGACTGTAAGGGATG	17	(ATG) 9	220–280	FAM (blue)

### Genetic data analysis

The sequences of the ITS rDNA and mtDNA *cox*2 obtained from all individuals were aligned using the software Clustal X v.2.0 (Larkin et al. 2007), with reference sequences from *C. osculatum* (s.l.) previously deposited in GenBank (Mattiucci et al. 2008). Phylogenetic analysis was carried out by Bayesian inference in MrBayes (Ronquist et al. 2012) based on the concatenated ITS rDNA and mtDNA *cox*2 sequences data. The best-fit substitution model was selected using the Bayesian Information Criterion (BIC) and the Akaike Information Criterion (AIC) as implemented in Jmodeltest (Darriba et al. 2012). A run of 1,000,000 iterations was completed, sampling the Markov Chains at interval of 100 generations. The first 20% of the trees were discarded as burn-in, and the remaining were visualized by FigTree v1.4.2 (http://tree.bio.ed.ac.uk/software/figtree/). Posterior probabilities were estimated and used to assess support for each branch in inferred phylogeny with probabilities where P = 95% being indicative of significant support (Reeder 2003). The phylogenetic tree was rooted by using *Phocanema decipiens* (s.s.) (MT347695) and *Ascaris suum* (X54253) as outgroups.

The parsimony network for the mtDNA haplotypes was inferred with the software PopART v.1.7 (Leigh and Bryant 2015) based on the TCS algorithm (Clement et al. 2000).

Analysis of molecular variance (AMOVA) on the mtDNA *cox*2 sequences and on SSRs-DNA loci was performed using 1000 permutations with the software ARLEQUIN v.3.5 (Excoffier and Lischer 2010). To investigate the occurrence of population structuring within the genetic datasets of mtDNA *cox*2 sequences and nDNA-SSRs loci, a Principal Component Analysis (PCA) was performed in R v.4.3.2 (R Core Team) using the packages *adegenet* (Jombart 2008) and *ade4* (Dray and Dufour 2007). The data, consisting of both sequences of mtDNA *cox*2 and standardized allele frequencies found at the SSRs-DNA loci, were first processed to obtain a genotype matrix. The number of principal components (PCs) was determined by examining the eigenvalues and the explained variance for each PC. The PCA results were visualized in a scatter plot, with the first two PCs (PC1 and PC2) plotted in the axes to highlight the main sources of variation within the dataset, using the package *ggplot2* implemented in R v.4.3.2 (Wickham 2016).

Further, a genetic distance matrix based on the mtDNA *cox*2 sequence data was calculated using the *ape* package (Paradis et al. 2004) implemented in R v.4.3.2 (R Core Team). The genetic distance values between all pairs of sequences were used to generate a heatmap, which was hierarchically clustered. Genetic differentiation among metapopulations was investigated based on pairwise fixation indices calculated with 1,000 permutations using ARLEQUIN v.3.5, on the genetic data sets acquired from both mtDNA *cox2* sequences and the SSRs-DNA loci.

The software Genepop (Rousset 2008) was used to verify the expected Hardy–Weinberg Equilibrium (HWE) at each SSR locus by exact test outcomes, adjusting the significance level (*p*) by the Bonferroni correction for multiple tests (Rice 1989). The inbreeding coefficient FIS was computed for each SSR locus to measure the deficiency (positive values) or excess (negative values) of heterozygotes at each locus, using the software ARLEQUIN v.3.5.

Genetic diversity inferred from the mtDNA *cox*2 data set was estimated at the parameters of: number of haplotypes (Nh), number of unique haplotypes (U), haplotype diversity (Hd), nucleotide diversity (π), average number of nucleotide differences (k), and number of polymorphic sites (S), by DnaSP v.5 (Librado and Rozas 2009). Genetic variability at the nDNA-SSRs loci was calculated in the same metapopulations of *C. osculatum* sp. B at the following parameters: percentage of polymorphic loci (P_99%_), alleles richness per locus (A), observed mean heterozygosity (H_O_), and expected mean heterozygosity (H_E_) by using ARLEQUIN v.3.5.

## Results

### Identification of *C. osculatum* (s.l.) specimens by ITS rDNA and mtDNA cox2

Out of the N = 388 historical *C. osculatum* (s.l.) specimens scored, high-quality sequences of the ITS region of rDNA and mtDNA *cox*2 gene locus were obtained for N = 208 specimens, allowing species identification. Meanwhile, all N = 214 individuals from the contemporary *C. osculatum* (s.l.) metapopulations considered in this study, were successfully sequenced at both gene loci (Table 3.). Thus, a 436 bp portion of the mtDNA *cox*2 gene was successfully obtained from a total of N = 422 specimens, including both historical and contemporary nematode specimens. Sequence analysis revealed that N = 418 of the sequenced nematodes matched at > 99% with the mtDNA *cox*2 sequences deposited in GenBank for *C. osculatum* sp. B. (accession numbers MT448512-19; EU477204); meanwhile, 4 L3 larvae collected from *Gadus morhua* matched at > 99% or 100% with the mtDNA *cox2* sequences deposited in GenBank for *C. osculatum* sp. A. (accession number EU477203) (Table 3.).

**Table 3:** Number of specimens assigned to the species *C. osculatum* sp. B or *C. osculatum* sp. A based on sequences analysis of the mtDNA *cox2* and the ITS rDNA, by sampling period, area and host species

		**mtDNA** *cox***2** **and ITS rDNA**			
**Code area**	**Host species**	**1985-86**		**2021-22**	
		*C. osculatum *sp. B	*C. osculatum *sp. A	*C. osculatum *sp. B	*C. osculatum *sp. A
BE	*Pagophilus groenlandicus* *Phoca vitulina*	24 12	- -	- -	- -
BS	*Pagophilus groenlandicus*	92	-	-	-
	*Gadus morhua*	-	-	60	-
FI	*Gadus morhua*	-	-	48	2
	*Pollachius virens*	-	-	10	-
JM	*Pagophilus groenlandicus*	80			
LO	*Gadus morhua*	-	-	62	2
SV	*Gadus morhua*	-	-	30	-
	Tot.	208	-	210	4

Similarly, all the N = 418 specimens matched at 100% with the ITS region of rDNA sequences deposited in GenBank for *C. osculatum* sp. B. Additionally, the 4 specimens assigned to *C. osculatum* sp. A based on mtDNA *cox*2, matched the ITS rDNA sequences of this species available in GenBank (accession numbers AJ250411/AJ250420 and AJ450410/AJ250419, respectively).

The phylogenetic analysis using Bayesian Inference (BI) (Suppl. Fig. S1) on both mtDNA *cox*2 and ITS rDNA sequences confirmed this finding, with all N = 208 historical and N = 210 current specimens clustering in a large clade with 100% posterior probability, alongside reference sequences of *C. osculatum* sp. B from GenBank (accession number EU477204) (Suppl. Fig. S1). This clade was distinctly separate from the similarly supported clade (100% posterior probability) in the BI analysis, formed by the 4 *C. osculatum* sp. A specimens found in the current samples, along with the reference specimen of *C. osculatum* sp. A previously deposited in GenBank (accession number EU477203), as well as from the other members of the *C. osculatum* (s.l.) complex (Suppl. Fig. S1). The sequences obtained at the ITS rDNA and mtDNA *cox*2 were deposited in GenBank under the accession numbers from PV715666 to PV715669 and from PV665656 to PV665659, respectively.

### Intraspecific genetic variation in mtDNA cox2 gene

The AMOVA analysis based on mtDNA *cox2* revealed that in both historical and contemporary *C. osculatum* sp. B metapopulations, most of the variance (over 99%) was significantly allocated within the analyzed metapopulations, rather than among them (Table 4A).

**Table 4:** **A**) Analysis of molecular variance (AMOVA) between and within metapopulations of the species *C. osculatum* sp. B, using genetic data sets obtained from mtDNA *cox*2. **B**) Analysis of molecular variance (AMOVA) between and within metapopulations of the species C. osculatum sp. B, using genetic data sets obtained from six SSRs loci (*Co42335*, *Co216165*, *Co97601*, *Co85484*, *Co210159* and *Co166121*)

			**Source of variation**	**d.f**	**Sum of squares**	**Variance components**	**Percentage of variation**
A)	mtDNA *cox*2	1985–86	Among populations	1	4.082	0.005	0.15
			Within populations	206	727.701	3.532	99.85
			Total	207	731.784	3.537	
		2021–22	Among populations	3	3.770	0.005	0.12
			Within populations	206	766.140	3.683	99.88
			Total	209	769.910	3.687	
B)	6 SSRs loci	1985–86	Among populations	1	1.728	0.028	1.20
			Among individuals within population	206	450.287	0.180	7.77
			Within individuals	208	391.000	2.210	91.03
			Total	415	843.015	2.418	
		2021–22	Among populations	3	18.210	0.030	1.05
			Among individuals within population	206	634.996	0.608	21.76
			Within individuals	210	417.000	2.152	77.18
			Total	419	1070.206	2.790	

**Fig. 2:**
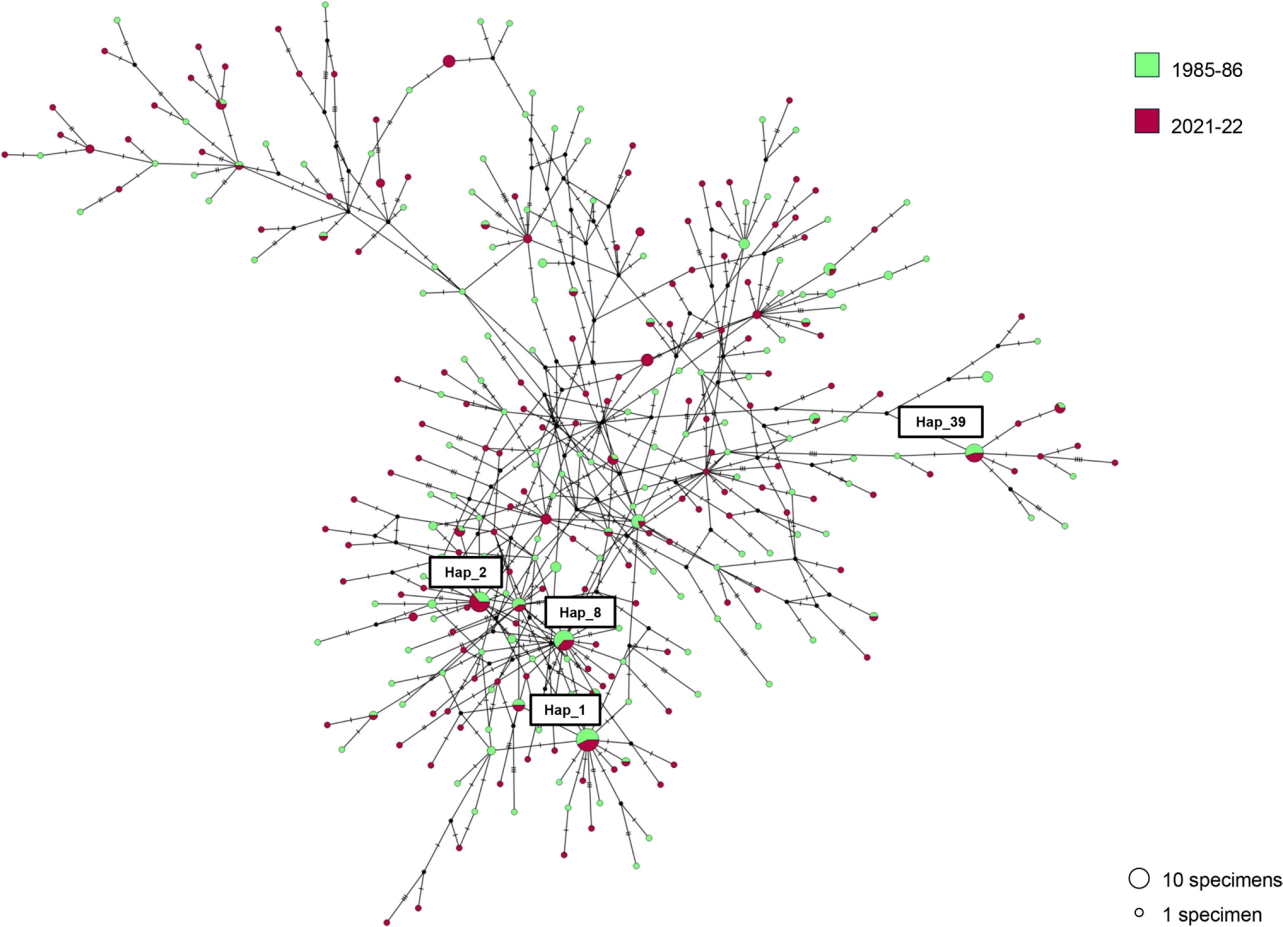
Statistical parsimony network inferred from the TCS algorithm (Clement et al. 2000) using the sequences of the mitochondrial gene *cox*2 of specimens of *Contracaecum osculatum* sp. B sampled between 1985–86 and 2021–22. Haplotypes observed in 1985–86 and in 2021–22 are coloured in light green and red, respectively. Each coloured circle represents a unique haplotype, and the size of circles are proportional to the number of individuals. The most frequent haplotypes shared over temporal scaled, are labelled

A star-like statistical parsimony network (TCS) (Fig. 2) carried out on the total specimens (N = 418) of the parasite species *C. osculatum* sp. B sequenced, was obtained. It illustrates the relationships among the haplotypes found in the two temporal periods (1985–86 and 2021–22). The network is predominantly characterized by numerous unique haplotypes, occurring as singletons, in both the temporal datasets. Specifically, out of a total of N = 312 haplotypes, 130 (approximately 42%) were singletons in the samples collected in 1985–86, and 138 (approximately 44%) were singletons in the samples from the 2021–22 period. Among the haplotypes found in adult specimens (1985–86), the most frequent ones occurred at a frequency of approximately 1.1%, corresponding to an average of two specimens per haplotype. Similarly, in the 2021–22 dataset, the nine most frequent haplotypes occurred at a frequency of approximately 1.2%, averaging three specimens per haplotype. Among the 25 haplotypes, shared across by the two sampling periods, 4 haplotypes (i.e. Hap_1, Hap_2, Hap_8, and Hap_39) were the most frequent, showing an average frequency of ≥ 3%; while, the remaining 21 shared haplotypes occurred at an average frequency of ≈1% each represented by, approximately, 4 specimens (Fig. 2).

**Fig. 3:**
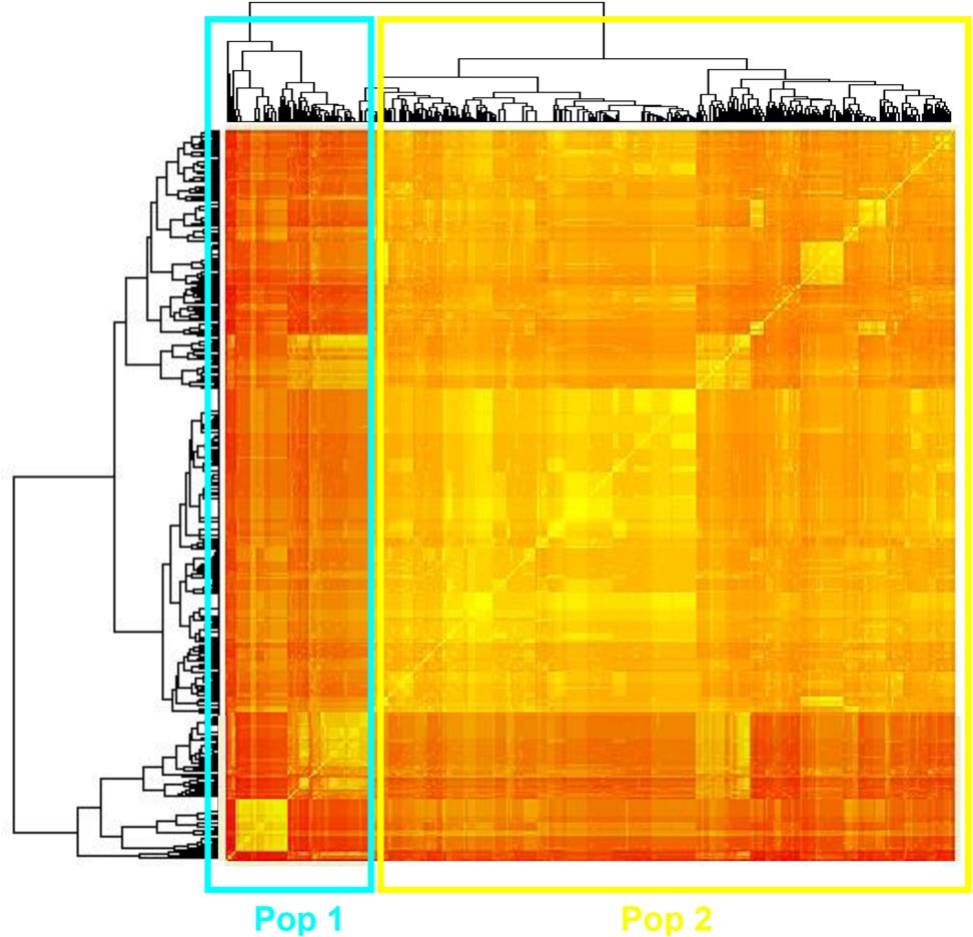
Heatmap displaying pairwise genetic distance values inferred from mtDNA *cox*2 sequences from specimens of *C. osculatum* sp. B (both historical and current samples). Rows and columns represent individual samples, clustered hierarchically based on their pairwise genetic distance values. The existence of two primary clusters is evident, likely corresponding to a substructuring of *C. osculatum* sp. B, here named as Pop 1 (light blue) and Pop 2 (yellow), respectively

At the intraspecific level, the heatmap analysis based on genetic distance data from mtDNA *cox*2 sequences, carried out on both historical and contemporary specimens (Fig. 3), revealed the existence of two main clusters, hereafter indicated as Pop 1 and Pop 2 (Fig. 3). A certain significant genetic differentiation was observed between Pop 1 and Pop 2, with an average *Fst* = 0.262 (*p* << 0.001). While, lower *Fst* values were observed at the intrapopulation level, i.e. within Pop 1 (*Fst* value close to zero, *p* = 0.405 ± 0.005), and Pop 2 (*Fst* value close to zero, *p* = 0.814 ± 0.004).

**Fig. 4:**
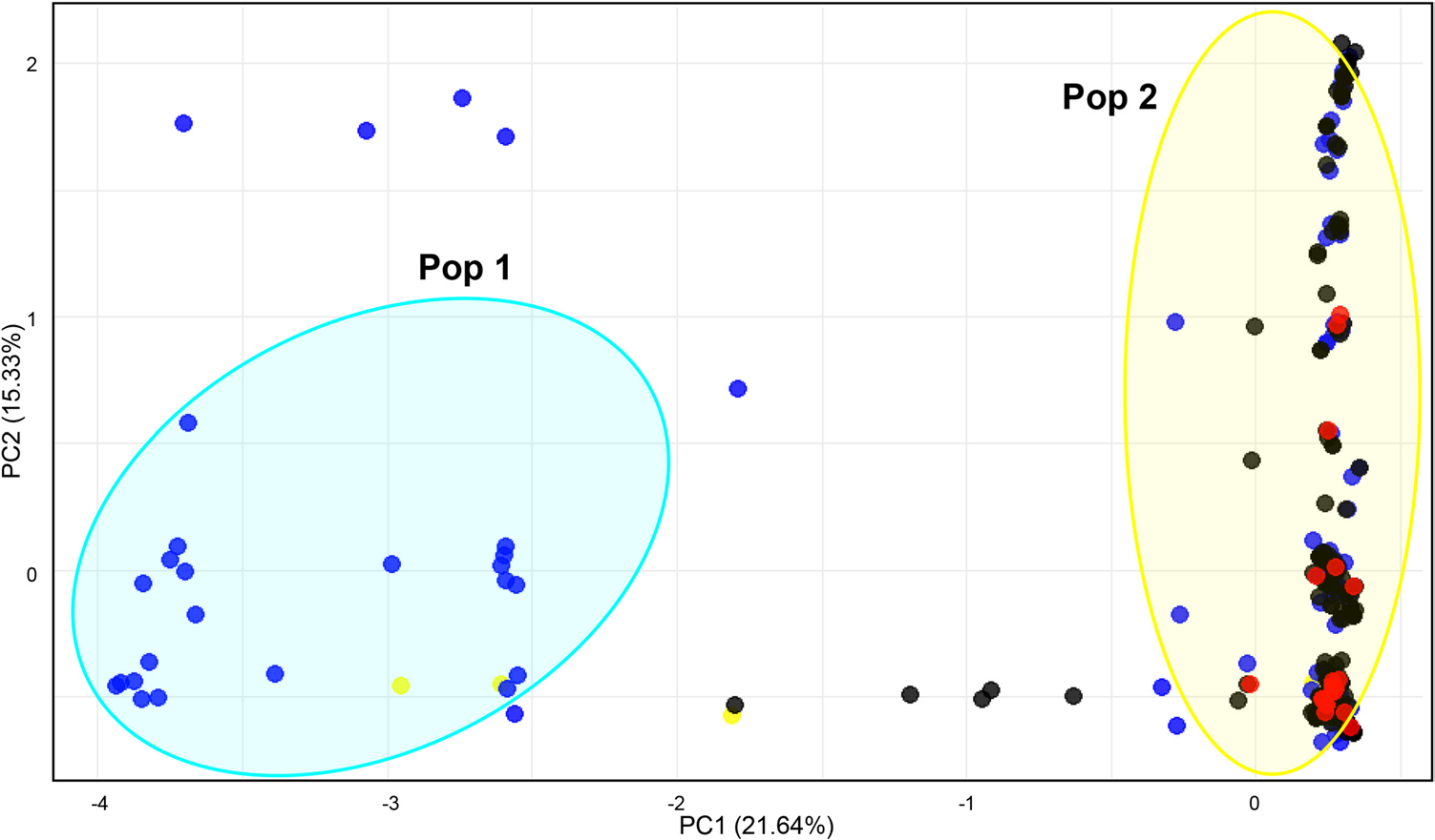
Scatter plot of the Principal Component Analysis (PCA) performed on mtDNA *cox2* sequences from adult and larval specimens of *C. osculatum* sp. B. The first two principal components explained 21.64% (PC1) and 15.33% (PC2) of the variance, with PC1 accounting for the separation between these two groupings. Colored clusters represent the Pop 1 (light blue) and Pop 2 (yellow), respectively. Red and yellow dots correspond to specimens of *C. osculatum* sp. B sampled from the harp seals and the harbour seal in the South Norwegian Sea, respectively, while blue dots represent specimens collected from harp seals the northern Barents-White Sea and the Jan Mayen area (see Table 1 for sampling locations). Black dots represent larval specimens sampled in fish hosts

The Principal Component Analysis (PCA) performed overall on both historical and contemporary samples, (Fig. 4), showed a consistent clustering pattern with the detection of the two main Pop 1 and Pop 2 clusters. Notably, the contemporary sample, formed by the larval specimens, predominantly clustered within Pop 2 (Fig. 4).

### Intraspecific genetic variation in the nuclear SSRs DNA loci

The total number of *C. osculatum* sp. B specimens genotyped at the seven scored SSRs DNA loci was N = 418, including historical (N = 208) and current samples (N = 210) (Table 3.). The AMOVA analysis, based on six nuclear SSRs-DNA loci, revealed that in both historical and current metapopulations of *C. osculatum* sp. B, most of the genetic variance was significantly allocated within the metapopulations (≈91% and ≈77%, respectively), rather than among them (Table 4B). Notably, a significant variation was found among individuals within the current metapopulations (≈22%) (Table 4B), with *FIT* = 0.17 (p < 0.05) (data not shown).

**Fig. 5:**
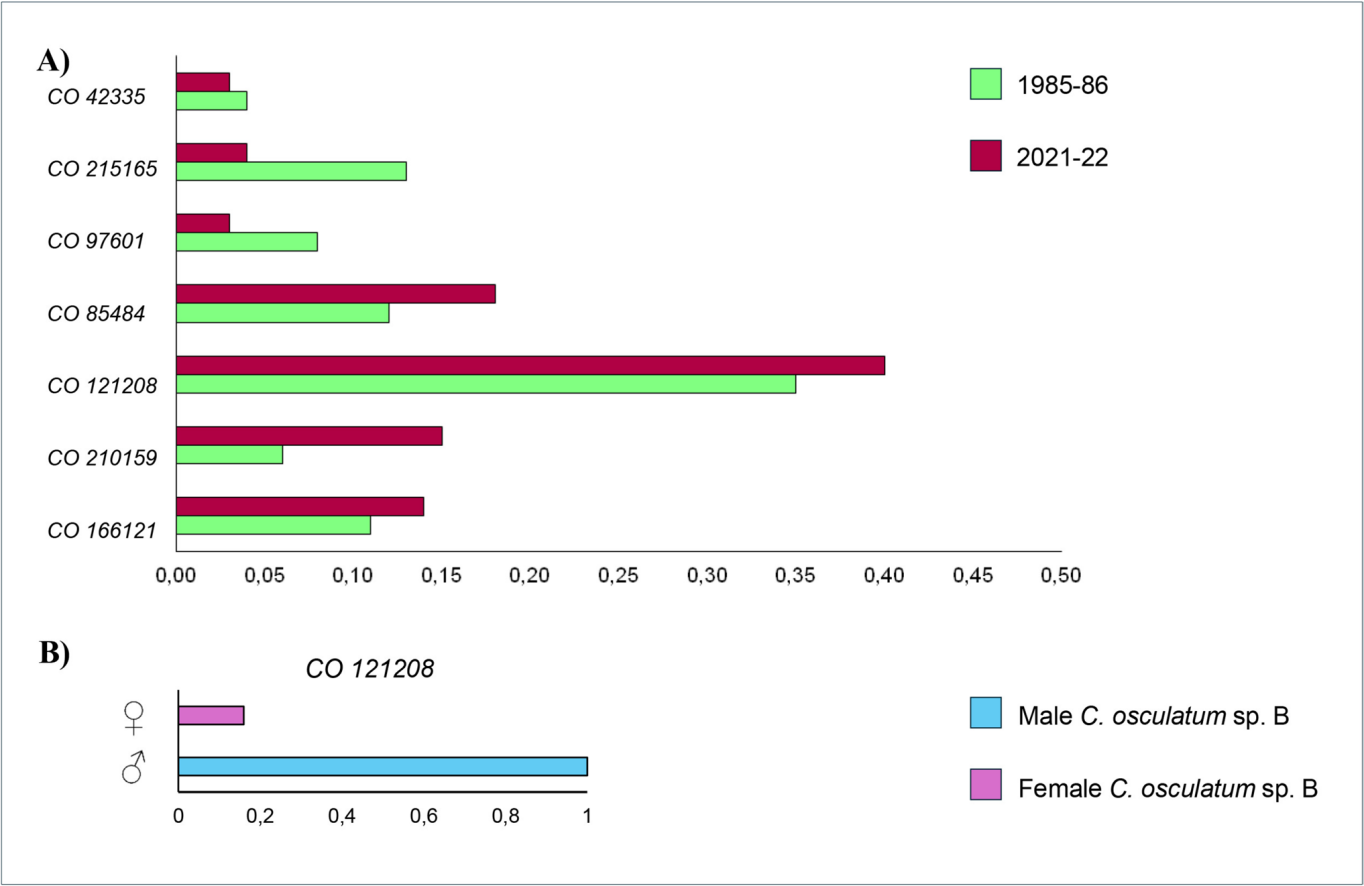
**A)** FIS calculated at the seven microsatellites loci studied in *C. osculatum* sp. B. Negative values indicate heterozygous excess while positive values indicate homozygous excess from that expected under Hardy–Weinberg Equilibrium (HWE), and **B**) FIS in male and female adult specimens at the sex-linked locus *Co121208*

The seven microsatellite loci scored were polymorphic in *C. osculatum* sp. B, with the total number of alleles ranging from 5 (observed at the locus *Co121208*) to 18 (found at the locus *Co210159)* (Table 5 A). Deviations from Hardy–Weinberg Equilibrium (HWE) at each locus were assessed in the selected populations of both species (Table 5A). Positive values of FIS indicated an excess of homozygous genotypes at the selected loci, while negative values suggested an excess of heterozygotes compared to the expected HWE (Fig. 5A). No significant deviations were found between observed (H_O_) and expected (H_E_) heterozygosity at six SSRs loci, in both historical and current *C. osculatum* sp. B populations, with the exception of thelocus *Co121208* (Table 5 A). This locus exhibited significant deviations from HWE overall in the parasite species, with an excess of homozygotes (Table 5 A), and a positive *FIS* value (Fig. 5A). Interestingly, when splitting the genotypes found at this locus, i.e. *Co121208,* found in the adult specimens of *C. osculatum* sp. B by sex, male worms (N = 18) were homozygous at this locus (*FIS* = 1) (Fig. 5B). In contrast, no significant departures from HWE were found (Table 5B) with a FIS value near to 0 (Fig. 5B), in the female specimens. These results suggest that the *Co121208* locus is sex-linked locus. Thus, it was excluded from the allele frequencies estimates formed by the contemporary larval population (Suppl. Table S1); while, in the case of historical adult sample, alleles frequencies were calculated only on adult females (N = 34) (Suppl. Table S1).

**Table 5:** **A)** Hardy Weinberg equilibrium observed at 7 SSRs DNA loci in the metapopulations of *C. osculatum* sp. B collected in the two different periods (1985–86 vs 2021–22). **B)** Hardy Weinberg equilibrium observed at the sex-linked locus, i.e. *Co121208*, in adult male and female specimens of *C. osculatum* sp. B sampled in 1985–86

A)			1985–86	2021–22
	**Locus**			
	*Co42335*	N	208	210
		*H*_*O*_	0.69	0.69
		*H*_*E*_	0.67	0.70
		*p* value	0.46	0.19
	*Co215165*	N	208	210
		*H*_*O*_	0.65	0.62
		*H*_*E*_	0.68	0.66
		*p* value	0.55	0.08
	*Co97601*	N	208	210
		*H*_*O*_	0.73	0.74
		*H*_*E*_	0.77	0.78
		*p* value	0.11	0.10
	*Co85484*	N	208	210
		*H*_*O*_	0.83	0.76
		*H*_*E*_	0.90	0.90
		*p* value	0.15	0.06
	*Co121208*	N	208	210
		*H*_*O*_	0.42	0.43
		*H*_*E*_	0.68	0.69
		*p* value	***	***
	*Co210159*	N	208	206
		*H*_*O*_	0.83	0.80
		*H*_*E*_	0.91	0.91
		*p* value	0.06	0.06
	*Co166121*	N	208	206
		*H*_*O*_	0.75	0.75
		*H*_*E*_	0.83	0.82
		*p* value	0.06	0.06
B)			**♀♀**	**♂♂**
	*Co121208*	N	32	18
		*H*_*O*_	0.56	0.00
		*H*_*E*_	0.66	0.65
		*p* value	0.43	***

N, number of individuals of *C. osculatum* sp. B studied at each SSRs locus; *H*_*O*_, observed heterozygosity; *H*_*E*_, expected heterozygosity; *p*, significance (*p* < 0.05) of the deviation from HWE expectation

**Fig. 6:**
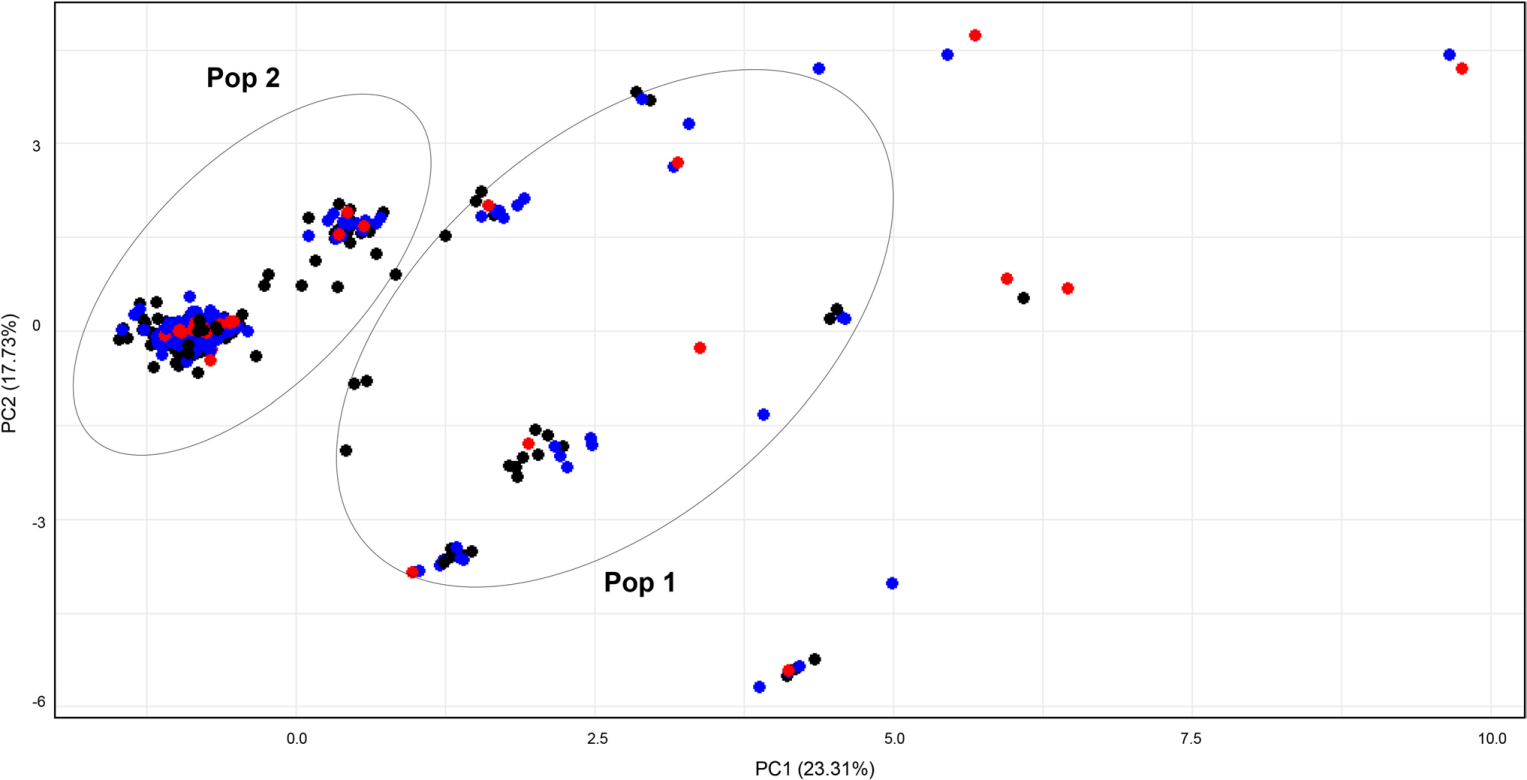
Scatter plot of the Principal Component Analysis (PCA) performed on the microsatellite (SSRs) dataset, including both adult and larval specimens of *C. osculatum* sp. PC1 explains 23.31% of the variance, while the Y-axis accounted for a 17.73%. Colors indicate specific host associations: red dots correspond to sampled from seals in the South Norwegian Sea, while blue dots represent specimens collected from the northern Barents-White Sea and the Jan Mayen area (see Table 1 for sampling locations). Black dots represent individuals sampled from fish hosts

The Principal Component Analysis (PCA) based on allele frequencies at the six SSRs DNA loci for the full dataset (N = 418 specimens), revealed a slightly separation along the X-axis, forming two distinct clusters indicated as well as Pop 1 and Pop 2 (Fig. 6). Slightly significant *Fst* value based on the SSRs-DNA loci was found between Pop 1 and Pop 2 (*Fst* = 0.04, *p* = 0.05 ± 0.235). No significant genetic differentiation was observed when comparing the specimens within the same Pop (data not shown). Similarly to the PCA based on mtDNA *cox*2, most of the contemporary specimens formed by L3 stage larvae, grouped within the Pop 2 (Fig. 6).

### Intraspecific genetic diversity at mitochondrial (mtDNA cox2) level

The estimates of genetic diversity parameters, based on the temporal datasets of mtDNA *cox*2 sequences obtained from *C. osculatum* sp. B specimens, are summarized in Table 6A. A total of N = 165 haplotypes were identified among specimens sampled between 1985–86. Out of these, 142 (approximately 86%) occurred once. Whereas, N = 172 haplotypes were identified from current samples dating from 2021–22, with 152 singletons (approximately 88%).

**Table 6:** **A)** Genetic variability estimates as inferred from mtDNA *cox*2 sequences analysis of metapopulations of *C. osculatum* sp. B sampled in 1985–86 and in 2021–22, defined by Principal Component Analysis (PCA), and overall. **B)** Genetic variability values at the parameters, as inferred from 6 polymorphic DNA SSRs loci analysis of metapopulations of *C. osculatum* sp. B sampled in 1985–86 and in 2021–22, defined by Principal Component Analysis (PCA), and overall

			Pop 1		Pop 2		Overall	Overall
			1985–86	2021–22	1985–86	2021–22	1985–86	2021–22
A)	**mtDNA *cox2***							
		N	36	40	172	170	208	210
		Nh (U)	30 (28)	32 (28)	136 (118)	140 (125)	165 (142)	172 (152)
		π ± SD	0.023 ± 0.001	0.021 ± 0.001	0.013 ± 0.001	0.013 ± 0.000	0.017 ± 0.000	0.017 ± 0.001
		Hd ± SD	0.979 ± 0.016	0.982 ± 0.012	0.994 ± 0.002	0.996 ± 0.002	0.996 ± 0.001	0.997 ± 0.001
		k	10.347	9.570	5.662	5.848	7.070	7.367
		S	64	60	86	99	107	113
B)	**6 SSRs loci**							
		N	36	40	172	170	208	210
		n	6	6	6	6	6	6
		P_99_	100	100	100	100	100.0	100.0
		A	8.0	9.3	13.5	11.5	13.5	11.5
		H_O_	0.72	0.73	0.74	0.66	0.73	0.63
		H_E_	0.79	0.81	0.80	0.80	0.80	0.80

N, number of considered sequences; Nh, number of haplotypes; U, number of unique haplotypes; π, nucleotide diversity; Hd, haplotype diversity; k, average number of nucleotide differences; S, number of polymorphic sites. N, number of analysed specimens; n, number of SSRs loci considered; P_99_, proportion of polymorphic loci at 99.0% criterion; A, average number of alleles per locus; H_O_, mean observed heterozygosity per locus; H_E_, mean expected heterozygosity per locus

Within the Pop 1, a total of 30 haplotypes of mtDNA *cox*2 were identified in 36 specimens of *C. osculatum* sp. B sampled during 1985–1986, whereas 136 haplotypes were detected in 172 individuals from the Pop 2 for the same period. Among the latter, 28 (approximately 93%) and 118 (approximately 87%) were singletons, respectively. In the contemporary parasite sample (2021–2022), 32 mtDNA *cox*2 haplotypes were identified in 40 specimens of *C. osculatum* sp. B from Pop 1, with 28 unique haplotypes (approximately 86%). Meanwhile, 140 haplotypes were detected in 170 individuals from Pop 2, of which 125 were singletons (approximately 89%) (Table 6 A).

The overall values of nucleotide diversity (π) and haplotype diversity (Hd) observed in the two temporal periods are shown in Table 6A. Lower nucleotide diversity (π) values were observed in the Pop 1 sampled in 2021–22 (π = 0.021 ± 0.001) in comparison with samples of years 1985–86 (π = 0.023 ± 0.001). Meanwhile, Pop 2 exhibited similar values (π = 0.013 ± 0.001) between the two sampling periods. Overall, the observed nucleotide diversity (π = 0.017 ± 0.001) was similar across both temporal samples of the parasite species (Table 6A).

### Intraspecific genetic variability at nuclear (SSRs-DNA loci) level

Estimates of genetic variability at six SSRs-DNA loci (excluding the sex-linked locus *Co121208*), based on the temporal and spatial dataset defined by the two clusters, Pop 1 and Pop 2, are presented in Table 6B. The six microsatellite loci were polymorphic across all the samples analyzed. The average number of alleles (A) ranged from A = 8.0 in Pop 1 (1985–86) to A = 13.5 in Pop 2 (1985–86). The lowest value of observed heterozygosity (H_O_) was recorded in Pop 2 sampled in 2021–22 (H_O_ = 0.66). Overall, the genetic variability value was higher in the samples collected between 1985–86 (H_O_ = 0.73) *versus* the current larval sample of *C. osculatum* sp. B from 2021–22 (H_O_ = 0.63). Similarly, the contemporary sample showed a slightly lower mean number of alleles (A = 11.5) in comparison with the 1985–86 samples (A = 13.5) (Table 6B).

**Fig. 7:**
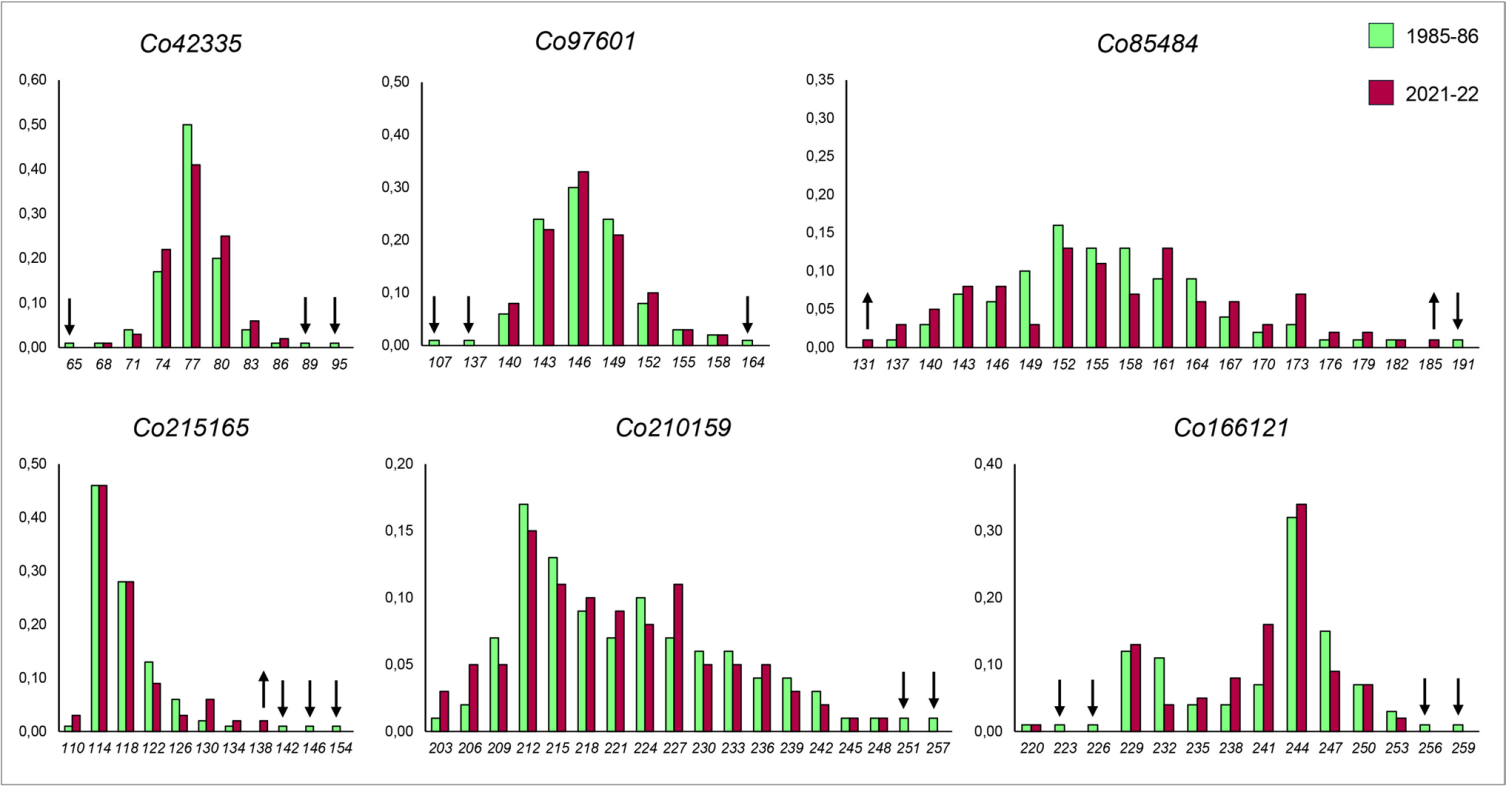
Histograms illustrating the allele frequencies observed at six microsatellite loci (excluding the sex-linked locus *Co121208*) in *C. osculatum* sp. B over temporal scale (1985–86 and 2021–22). Each bar represents the frequency (Y-axis) of an allele at a given locus (X-axis). ↓ indicates apparent lost alleles, while ↑ indicates new observed alleles

Histograms in Fig. 7 illustrate the overall temporal variation in standardized allelic frequencies of *C. osculatum* sp. B by comparing those from historical and current metapopulations. Notably, several rare alleles present in the SSRs DNA loci in the historical population were missing in the current population (Fig. 7). For example, alleles *251* and *257* at the locus *Co210159*, and alleles *223, 226, 256,* and *259* at the locus *Co166121* are missing. Additionally, other SSRs loci showed a lower frequency in the current population compared to the historical sample (Fig. 7). Conversely, new rare alleles appear to be present in the current populations—such as those found at the loci *Co215165* (allele *138*) and *Co85484* (alleles *131* and *185*)—which were not observed in the historical sample (Fig. 7).

## Discussion

This study provides the first analysis of population genetic structure and the estimation of genetic variability over temporal scale of three-decades for the Arctic anisakid sibling species *C. osculatum* sp. B. Most population genetics studies of marine parasites infer microevolutionary mechanisms based on data from a single time point (Mattiucci et al. 2018; 2025a). A recent analysis of population genomics performed on seal lice has helped to elucidate the evolutionary history speculated by its seal hosts in the Baltic and Arctic Sea waters (Sromek et al. 2024). However, to directly observe dynamic processes, it is crucial to perform temporal population surveys. So far, few molecular studies on parasites and vectors have analyzed population genetic structure across multiple temporal points. These studies have applied various markers, including SSRs-DNA loci and mitochondrial DNA, and primarily focus on nematode model species such as *Caenorhabditis elegans* (Barrière and Félix 2007) or on the malaria parasite *Plasmodium falciparum* (Niang et al. 2017; Huang et al. 2018) and its mosquito vectors (Lukindu et al. 2018). Generally, studies on the population genetic structure over a temporal scale are lacking for marine parasites (rev. in Mattiucci et al. 2025a). Previous population genetic analyses of anisakid nematodes, including comparisons with abundance estimates over time, were performed in the Antarctic members of *C. osculatum* (s. l.), specifically *C. osculatum* sp. D and *C. osculatum* sp. E. This analysis revealed that both the high genetic variability and parasitic load (as a proxy for population size) of these Antarctic worms remained stable over two decades (Mattiucci et al. 2015). The finding was attributed to the large population size of both Antarctic seals (*Leptonychotes weddellii*) and fish hosts which serve as definitive and intermediate/paratenic hosts for the parasites, respectively. It was thus suggested that the stability of the trophic web connecting these hosts contributes to maintain a high parasite transmission rate in the Antarctic region (Mattiucci et al. 2015).

### Novel nuclear polymorphic loci in *C. osculatum* sp. B

This study highlights the utility of polymorphic loci in population genetics by combining mitochondrial and nuclear markers to capture fine-scale patterns of parasite genetic variation, providing a comprehensive view of population structure, even across a temporal scale. In particular, the development and validation of SSRs DNA markers for *C. osculatum* sp. B represents a significant methodological advancement. The use of microsatellites in anisakid parasites has proven to be an invaluable tool for understanding their population dynamics, genetic structure, and evolutionary processes. Previous studies have been successfully applied SSRs DNA loci to investigate various genetic aspects of marine parasites, providing insights into their population structure, genetic diversity, and phenomena such as hybridization and introgression. Jossart et al. (2017) used ten microsatellite loci to explore patterns of population structure in the parasitic species *Dissodactylus primitivus* and its host, the heart urchin. The population genetic structure of *Lepeophtheirus salmonis*, an ectoparasite of Atlantic and Pacific salmonids, has been studied through the analysis of microsatellite DNA variation among wild and farmed hosts (Todd et al. 2004). Additionally, Criscione and Blouin (2007) employed microsatellite analysis of trematode parasites to trace the origin of the steelhead trout *Oncorhynchus mykiss*. The application of SSRs DNA markers in parasitic nematodes has been relatively limited (Betson et al. 2011; Patrelle et al. 2014; Rabelo et al. 2017; Greeff et al. 2018). In the case of anisakid nematodes, SSRs DNA loci were successfully developed and validated in the three species of the *Anisakis simplex* (s. l.) complex (Mladineo et al. 2017; Mattiucci et al. 2019; Bello et al. 2020). These loci have proven to be of diagnostic value in identifying the parental genome of the three species and correctly classifying hybrid categories and to detect mitochondrial introgression between *A. pegreffii* and *A. simplex* (s. s.) (Mattiucci et al. 2025b), as well as F1 hybrids between *A. pegreffii* and *A. berlandi* (Bello et al. 2021).

In the present study, seven loci were successfully validated through robust amplification from individual worms, providing clear and unambiguous genotype detection across a high number of specimens (N = 418) of *C. osculatum* sp. B. Notably, in *C. osculatum* sp. B, the microsatellite markers exhibited a high level of polymorphism, with expected heterozygosity (H_E_) values ranging from H_E_ = 0.67 at the locus *Co215165* to H_E_ = 0.91 at the locus *Co210159*, showing an overall average of H_E_ = 0.78. The average number of alleles per locus was A = 11.1, slightly higher than the value of 10.8 observed for the microsatellites developed for the *A. simplex* (s.s.) complex (Mattiucci et al. 2019; Bello et al. 2020, 2021). The high polymorphism observed suggests that the microsatellites developed in this study are valuable tools for fine-scale genetic studies, including assessments of population differentiation, gene flow, genetic diversity, and the detection of genetic erosion occurrence in the gene pools of these marine anisakid nematodes.

Thus, these markers can thus be used in future population genetics studies of this species, sampled in other geographical areas, as well as in other species of the *C. osculatum* (s. l.) complex from their range of distribution.

Additionally, an interesting finding in the SSRs DNA loci was the discovery of a sex-linked locus in *C. osculatum* sp. B. Sex-linked loci have been previously reported in other anisakid nematodes, such as species within the *A. simplex* (s.l.) complex (Mattiucci et al. 2019, 2025b; Bello et al. 2020, 2021). Sex-linked SSR DNA loci have also been identified in *A. lumbricoides*, where they may constitute around 20% of its genome. According to Mattiucci et al. (2019, 2025b), these loci represent approximately 33% of the SSRs DNA loci in *A. simplex* (s.l.). The discovery of sex-linked SSR markers in anisakid nematodes could be instrumental for accurate gender assignment at any life-history stage of these parasites.

### Population genetic structure of *C. osculatum* sp. B

**Fig. 8:**
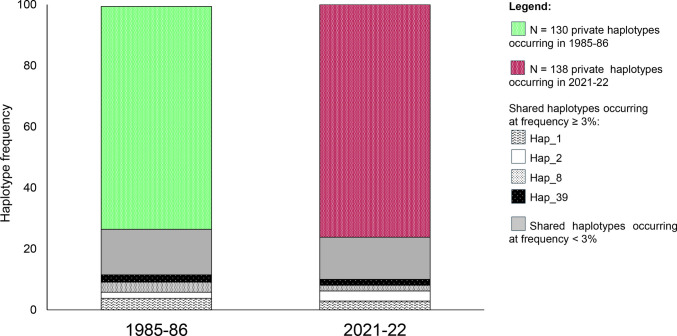
Temporal variation in haplotype frequencies observed in the two datasets (1985–86 and 2021–22). The most frequent haplotypes shared over temporal scale occurring at frequency > 3%, i.e., Hap_1, Hap_2, Hap_8, Hap_39, are represented (see Legend for the specific colour patterns). Haplotypes shared over temporal scale occurring at frequency < 3% are coloured in grey. Private haplotypes occurring at frequency < 3% in 1985–86 and in 2021–22 are coloured in light green and red, respectively

The Arctic member C*. osculatum* sp. B appears a panmictic species, as shown by the star-like haplotype network (Fig. 2). At the intraspecific level, the heatmap analysis and PCA analyses based on mtDNA *cox2* indicated the existence of a slight genetic subpopulation structuring with a certain significant level of genetic differentiation (*Fst* value). This finding suggests the presence of intraspecific metapopulations structure in *C. osculatum* sp. B in the study area. The degree of genetic differentiation found at the mitochondrial level is likely due to the difference in the rare and unique haplotypes found in the parasite species between the historical and contemporary samples (Figs. 2 and 8). Similar level of intraspecific genetic differentiation at the mitochondrial level has been previously observed among conspecific populations of other anisakid species, which maintain a high gene flow between their metapopulations by the vagility of the hosts involved in their life-cycle (Mattiucci and Nascetti 2008; Mattiucci et al. 2025a). It has been suggested that population genetic structure of definitive hosts participating in the life-cycle of anisakids may be a driver in shaping the population structure of their endoparasites (Mattiucci et al. 2025a). Interestingly, the existence of these two subpopulations of the parasite species appears to be linked to the intraspecific population genetic structuring of its main definitive host in the study area, the harp seal (*Pagophilus groenlandicus*). Indeed, previous studies on the population genetic structure of harp seal populations, inferred from mitochondrial *cytb* gene sequences, revealed significant genetic differentiation between Northwest and Northeast Atlantic populations (Perry et al. 2000). Additionally, distinct populations of the harp seal have been identified based on their breeding grounds (i.e., Northwest Atlantic, Greenland Sea, and White Sea–Barents Sea) (Folkow et al. 2004; Nordøyet al. 2008). The population genetic structure of *C. osculatum* sp. B in the historical sample, appears to mirror that of its definitive hosts, primarily the harp seal, from which the adults were collected. This finding may result from host-parasite microevolutionary co-phylogeographical events in the Arctic Sea waters. Co-phylogenetic patterns between pinniped hosts and their anisakid endoparasites in the genus *Contracaecum* have been proposed based on the comparison of phylogenetic analyses of the two interacting groups (pinnipeds and *Contracaecum* spp. maturing in seal hosts) (Mattiucci and Nascetti 2008). These patterns suggest that the anisakids have co-evolved with their definitive hosts throughout evolutionary history. Thus, the harp seal populations, from which the parasite was collected, may be a source of genetic variation, as indicated by the clustering patterns here observed, while the main fish hosts may contribute to maintaining high gene flow between the populations of *C. osculatum* sp. B.

Indeed, the contemporary populations of *C. osculatum* sp. B analyzed, consisting of larval specimens sampled from fish hosts — primarily *Gadus morhua* (cod) — did not show clear distinctiveness between two clusters. Most of them likely clustered within the Pop 2 (Fig. 4). It would be reasonable to hypothesize that the fish hosts of the parasite species likely play an important role in maintaining the gene flow between the metapopulations of *C. osculatum* sp. B in Arctic waters. This could be the consequence of their high mobility and dispersion in the Arctic/Sub-arctic ecosystems. Thus, the role of fish hosts in facilitating genetic homogenization should not be overlooked. The panmixia observed in this study in *C. osculatum* sp. B (Fig. 2) likely reflects the ecological connectivity driven by trophic transmission, with fish serving as a driver of genetic exchange among geographically dispersed definitive host populations in Arctic and sub-Arctic waters. The gene flow between the parasite metapopulations may be due to the long-distance migratory behaviour of the North East Atlantic Cod "ecotype" (indicated as NEAC) (Dahle et al. 2018), which occasionally migrates northward (Bogstad 2023).

Overall, these findings suggest that the population genetic structure of *C. osculatum* sp. B might be shaped by both seal definitive and fish intermediate/paratenic host population structures. This supports the idea that this parasite species may also reflect the structure of the trophic web in Arctic Sea waters, including seals and fish as key hosts in its life cycle.

### Genetic variability over a temporal scale in *C. osculatum* sp. B

**Fig. 9:**
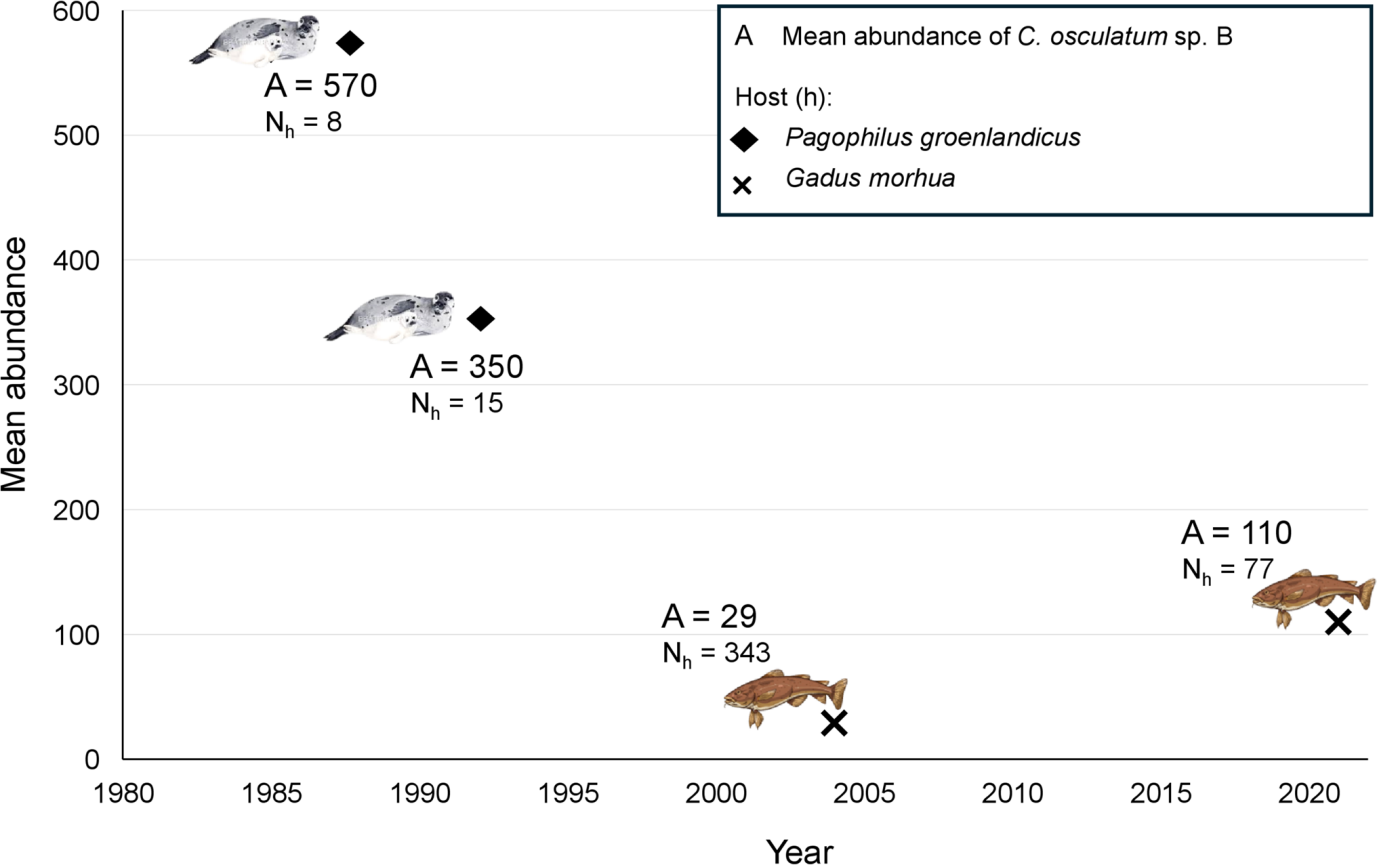
Mean abundance of *C. osculatum* sp. B in Arctic and sub-Arctic populations of *Pagophilus groenlandicus* (black diamonds) and *Gadus morhua* (crosses), showing demographic variations likely occurred in the past three decades, according to our repository data concerning seals infection, and records by Perdiguero-Alonso et al. (2008) and Levsen et al. (2022) concerning fish infection

The analysis of mtDNA *cox*2 revealed substantial changes in genetic variability over the temporal scale. In the historical sample, a high number of unique haplotypes were identified, many of which were not found in the contemporary samples. Specifically, the two temporal samples exhibited a high proportion of unique haplotypes, 86% and 88%, respectively. This pattern is further supported by the TCS haplotype network (Fig. 2) and illustrated by histograms representing haplotype frequency variations over time, which also highlight a limited number of shared variants between the two temporal samples considered here (Fig. 8). The apparent absence of certain rare haplotypes in the contemporary sample, compared to the historical one, may indicate a loss of genetic polymorphism due to genetic drift, driven by fluctuations in host populations or environmental changes that directly affect the free-living larval stages. Conversely, the appearance of new haplotypes at the mtDNA *cox*2 locus, such as those observed in the current samples, suggests ongoing microevolutionary processes. These findings suggest a dynamic process of haplotype turnover, where rare haplotypes may have been lost over time, only to be replaced by new variants (Fig. 8). This turnover could help explain the overall similarity in nucleotide and haplotype diversity between the two temporal datasets. It can be speculated that this process reflects demographic shifts in the parasite population, potentially driven by fluctuations in both its intermediate and definitive hosts (Fig. 9). These processes, potentially linked to the recent demographic expansion of the parasite species evidenced by an increased abundance of infection in intermediate hosts (Levsen et al. 2022), may promote the occurrence of new haplotypes. In this regard, a demographic expansion of the cod population in recent years has been reported (Kjesbu et al. 2014; Rose and Rowe 2015; ICES 2021).

Additionally, this may also be related to the potential immigration of *C. osculatum* sp. B, acquired by its hosts from other Arctic regions not investigated here, such as the Northwest Atlantic, where the species is also present (Nascetti et al. 1993). Such immigration could explain the occurrence of the new haplotypes detected in this study. Interestingly, in support of this hypothesis, some haplotypes identified in the present study in the contemporary populations were detected in recent analysis, from the Northwest Atlantic, specifically in Canadian waters (Severin et al. 2020) (GenBank accession numbers: MT448515 and MT448519). In contrast to the mitochondrial gene, analysis of the SSRs DNA loci showed a lower level of differentiation between the two temporal data sets (*Fst* close to zero). This may be due to the generally lower mutation rate of microsatellites compared to the mitochondrial DNA, as well as to their biparental inheritance, which can obscure the patterns of genetic differentiation observed in mitochondrial DNA.

At nuclear level, the reduction of the observed heterozygosity (H_O_) and average number of alleles (A), as well as the loss of rare alleles along with a lower frequency distribution of certain alleles (Fig. 7) observed in the contemporary metapopulations Pop2 of the parasite species, with respect to the historical sample, provides further evidence of a decline in the parasite's genetic variability. Indeed, losing of rare alleles has been reported as the first phenomenon observed during population bottlenecks (Gatto-Almeida et al. 2022). Taken together, these observations indicate possible genetic erosion events in the contemporary population sample, suggesting that *C. osculatum*sp. B populations examined in this study may have undergone a demographic decline over the past 35–40 years (Fig. 9).

However, the apparent incongruence between the observed decline in unique genetic polymorphism at both mitochondrial and nuclear level in *C. osculatum* sp. B over the past 35–40 years, alongside the emergence of novel haplotypes, may be linked to fluctuations in host population size and subsequent changes in parasite abundance values (Fig. 9). Throughout this period, both seal and fish populations—critical hosts in the parasite’s life cycle—have indeed undergone periods of decline due to viral outbreaks and overfishing, followed by recoveries driven by conservation efforts and fishery management. Given the parasite's reliance on trophic transmission and high host preference (Mattiucci and Nascetti 2008), reduction in host abundance (Fig. 9), could had likely influenced population size and genetic diversity of their endoparasites nematodes.

Indeed, in the studied area, marine resource overexploitation and environmental shifts, including climate change, have contributed to habitat degradation and fluctuations in the key fish stocks that serve as intermediate or paratenic hosts for *C. osculatum* sp. B, such as Arctic cod (*Gadus morhua*) and capelin (*Mallotus villosus*) (Bundy et al. 2009; Gjøsæter et al. 2009). Similarly, pinniped populations—its definitive hosts—have experienced declines, including severe phocine distemper virus (PDV) epidemics that killed over 23,000 harbour seals in 1988 and 30,000 in 2002 (Härkönen et al. 2006), followed by substantial recoveries (Brasseur et al. 2018). Harp seals have also faced population fluctuations due to reduced seasonal sea ice, a crucial breeding platform (Johnston et al. 2012). This recovery has likely contributed to the resurgence of parasite populations (Olsen et al. 2007), suggesting that shifts in host dynamics play a key role in shaping the genetic variability of *C. osculatum* sp. B. The acquisition of new mitochondrial and nuclear variants in *C. osculatum* sp. B populations might align with reports of demographic expansion in Arctic seals and fish populations, as a consequence of conservation measures' implementation. These measures may have facilitated the parasite’s demographic recovery after an initial decline, promoting the spread of novel haplotypes at the mtDNA *cox*2 locus and new alleles at nuclear SSRs DNA loci. Therefore, the observed genetic changes in the parasite species likely reflect significant perturbations within the Arctic food web, driven by human-induced alterations.

A comparable pattern has been observed in the Baltic Sea, where increasing abundance of *C. osculatum* (s. s.) in both cod and grey seals—its primary definitive hosts—coincide with a rapid expansion of seal populations (Haarder et al. 2014; Mehrdana et al. 2014; Nadolna and Podolska 2014; Zuo et al. 2016; Buchmann 2023; Kuman et al. 2025). However, the overall high level of heterozygosity across the SSRs DNA loci and the haplotype variation estimates indicates that these metapopulations of *C. osculatum* sp. B still maintain its high genetic diversity. This characteristic is crucial, as it may help natural populations to cope with and adapt to environmental changes (Birader 2023).

The observations here acquired align with existing literature suggesting that climate-driven changes in Arctic marine ecosystems, such as rising sea temperatures influencing host distribution (Gerland et al. 2023), might also act as selective pressures on parasite populations. For example, warmer temperatures can directly affect the survival and transmission efficiency of parasite larval stages, while shifts in host migration patterns may influence the genetic composition of parasite populations (Davidson et al. 2011).

## Conclusions

This study provides new insights into the population genetic structure of *C. osculatum* sp. B over a temporal scale spanning several decades, emphasizing the role of definitive seal and intermediate/paratenic fish host populations demography in shaping the parasite's genetic variability. The findings highlight how the genetic diversity and abundance of this trophically transmitted parasite can serve as a baseline for monitoring Arctic trophic-webs stability.

Given that anthropogenic effects and environmental perturbations due to climate change are particularly evident in the Arctic ecosystem (Steiner et al. 2021; Deb and Bailey 2023; Gerland et al. 2023), this study underscores the value of a marine anisakid worm as an ecological indicator of how high-latitude host-parasite systems may respond to human impacts. Monitoring this parasite species in the Arctic-Boreal region acquires major importance also in consideration of the possible zoonotic role of the species of *C. osculatum* s.l. complex (EFSA, 2024).

Future research should integrate parasites genetic data with biotic factors, such as additional host species to explore different trophic interactions, as well as abiotic environmental variables such as ocean temperature rise, reduced sea-ice extent and changes in water chemistry. This will help clarify the relationship between anthropogenic stressors and parasite populations, leading to a better understanding of the ecological and evolutionary factors driving genetic diversity in the *C. osculatum* (s.l.) species complex.

Investigating the host-parasite co-phylogeography between the *C. osculatum* (s.l.) species complex and their specific definitive hosts will provide valuable insights into the historical dynamics and microevolutionary events occurred in these parasites inhabiting Arctic-Boreal waters.

## Supplementary Information

Below is the link to the electronic supplementary material.

ESM 1 (TIF. 7.66 MB)

ESM2 (DOC.155 KB)

## Data Availability

Data is provided within the manuscript.
