# Supplementary figures and images for: Spatio-temporal analysis of genetic diversity in the sibling species Contracaecum osculatum sp. B: a tool for monitoring trophic-web dynamics in Arctic Sea waters
Source: Parasitol Res. 2025 Nov 29;124(12):147. doi: 10.1007/s00436-025-08561-6 (PMC12665636; doi:10.1007/s00436-025-08561-6)

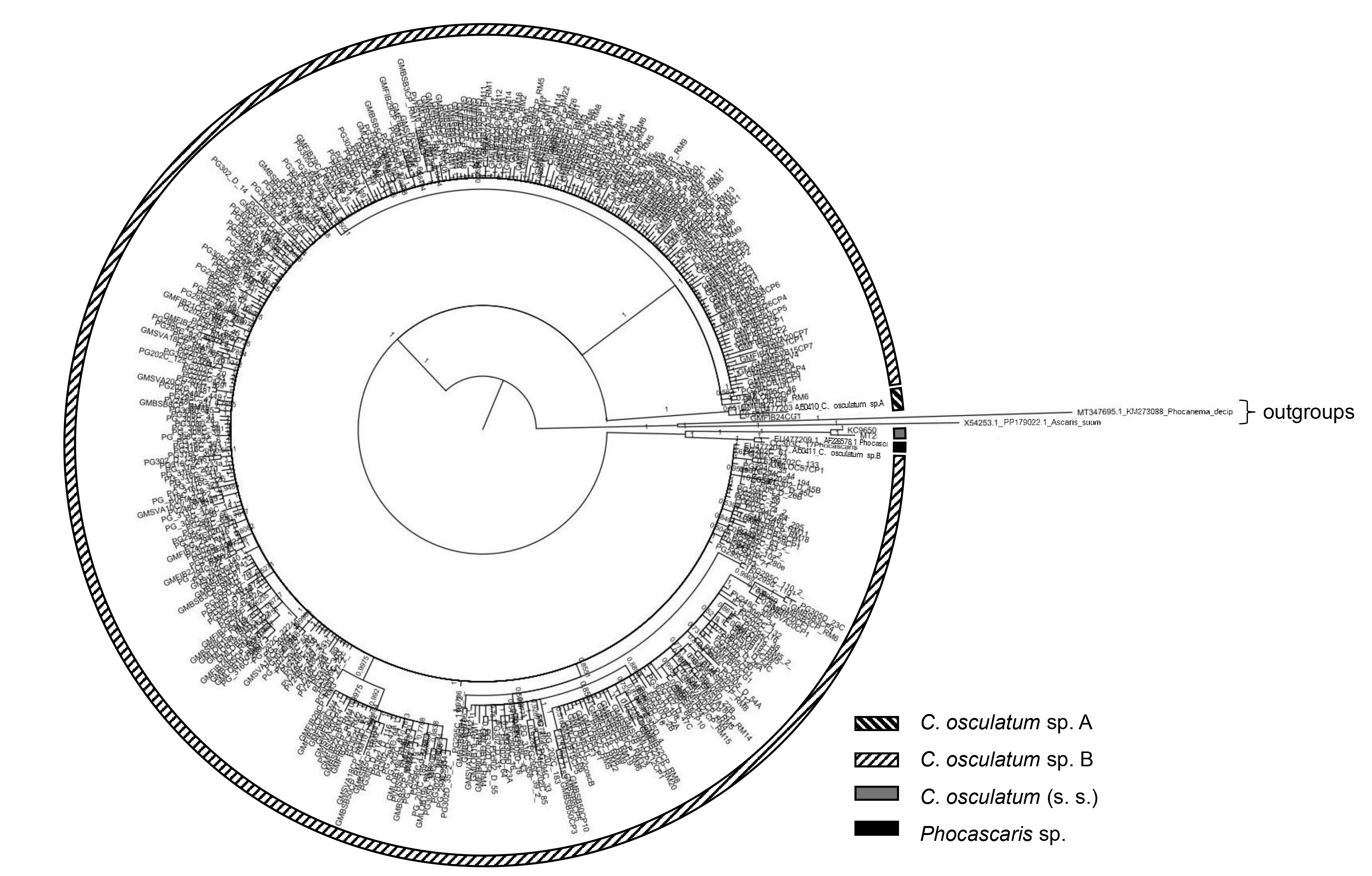

Supplement: Supplementary file 1 — (TIF. 7.66 MB) [file 436_2025_8561_MOESM1_ESM.tif]
