# Supplementary material for: Spatio-temporal analysis of genetic diversity in the sibling species Contracaecum osculatum sp. B: a tool for monitoring trophic-web dynamics in Arctic Sea waters
Source: Parasitol Res. 2025 Nov 29;124(12):147. doi: 10.1007/s00436-025-08561-6 (PMC12665636; doi:10.1007/s00436-025-08561-6)
Supplement: Supplementary file 2 — (DOC.155 KB) [file 436_2025_8561_MOESM2_ESM.doc]

**Supplementary Table 1.** Allele frequencies, occurring at frequency ≥0.01, observed at six SSRs loci tested in populations of *C. osculatum* sp. B sampled in the two different temporal periods (1985-86 and 2021-22). As regards the sex-linked locus (i.e. *Co121208*) the most reliable estimate of allele frequencies was calculated in adult population according to the sex-linked genetic model estimate, assuming: (i) the hemizygosity of the males at that locus; (ii) their adult female counterparts, as biallelic at the sex-linked locus.

|  |  | **1985-86** |  | **2021-22** |
| --- | --- | --- | --- | --- |
| **Locus** | **Alleles** |  |  |  |
| *Co42335* | *65* | 0.01 |  | - |
|  | *68* | 0.01 |  | 0.01 |
|  | *71* | 0.04 |  | 0.03 |
|  | *74* | 0.17 |  | 0.22 |
|  | *77* | 0.50 |  | 0.41 |
|  | *80* | 0.20 |  | 0.25 |
|  | *83* | 0.04 |  | 0.06 |
|  | *86* | 0.01 |  | 0.02 |
|  | *89* | 0.01 |  | - |
|  | *95* | 0.01 |  | - |
|  |  |  |  |  |
| *Co215165* | *110* | 0.01 |  | 0.03 |
|  | *114* | 0.46 |  | 0.46 |
|  | *118* | 0.28 |  | 0.28 |
|  | *122* | 0.13 |  | 0.09 |
|  | *126* | 0.06 |  | 0.03 |
|  | *130* | 0.02 |  | 0.06 |
|  | *134* | 0.01 |  | 0.02 |
|  | *138* | - |  | 0.02 |
|  | *142* | 0.01 |  | - |
|  | *146* | 0.01 |  | - |
|  | *154* | 0.01 |  | - |
|  |  |  |  |  |
| *Co97601* | *107* | 0.01 |  | - |
|  | *137* | 0.01 |  | - |
|  | *140* | 0.06 |  | 0.08 |
|  | *143* | 0.24 |  | 0.22 |
|  | *146* | 0.30 |  | 0.33 |
|  | *149* | 0.24 |  | 0.21 |
|  | *152* | 0.08 |  | 0.10 |
|  | *155* | 0.03 |  | 0.03 |
|  | *158* | 0.02 |  | 0.02 |
|  | *164* | 0.01 |  | - |
|  |  |  |  |  |
| *Co85484* | *131* | - |  | 0.01 |
|  | *137* | 0.01 |  | 0.03 |
|  | *140* | 0.03 |  | 0.05 |
|  | *143* | 0.07 |  | 0.08 |
|  | *146* | 0.06 |  | 0.08 |
|  | *149* | 0.10 |  | 0.03 |
|  | *152* | 0.16 |  | 0.13 |
|  | *155* | 0.13 |  | 0.11 |
|  | *158* | 0.13 |  | 0.07 |
|  | *161* | 0.09 |  | 0.13 |
|  | *164* | 0.09 |  | 0.06 |
|  | *167* | 0.04 |  | 0.06 |
|  | *170* | 0.02 |  | 0.03 |
|  | *173* | 0.03 |  | 0.07 |
|  | *176* | 0.01 |  | 0.02 |
|  | *179* | 0.01 |  | 0.02 |
|  | *182* | 0.01 |  | 0.01 |
|  | *185* | - |  | 0.01 |
|  | *191* | 0.01 |  | - |
|  |  |  |  |  |
| *Co121208* | *160* | 0.18 |  | na |
|  | *163* | 0.44 |  | na |
|  | *166* | 0.33 |  | na |
|  | *169* | 0.05 |  | na |
|  |  |  |  |  |
| *Co210159* | *203* | 0.01 |  | 0.03 |
|  | *206* | 0.02 |  | 0.05 |
|  | *209* | 0.07 |  | 0.05 |
|  | *212* | 0.17 |  | 0.15 |
|  | *215* | 0.13 |  | 0.11 |
|  | *218* | 0.09 |  | 0.10 |
|  | *221* | 0.07 |  | 0.09 |
|  | *224* | 0.10 |  | 0.08 |
|  | *227* | 0.07 |  | 0.11 |
|  | *230* | 0.06 |  | 0.05 |
|  | *233* | 0.06 |  | 0.05 |
|  | *236* | 0.04 |  | 0.05 |
|  | *239* | 0.04 |  | 0.03 |
|  | *242* | 0.03 |  | 0.02 |
|  | *245* | 0.01 |  | 0.01 |
|  | *248* | 0.01 |  | 0.01 |
|  | *251* | 0.01 |  | - |
|  | *257* | 0.01 |  | - |
|  |  |  |  |  |
| *Co166121* | *220* | 0.01 |  | 0.01 |
|  | *223* | 0.01 |  | - |
|  | *226* | 0.01 |  | - |
|  | *229* | 0.12 |  | 0.13 |
|  | *232* | 0.11 |  | 0.04 |
|  | *235* | 0.04 |  | 0.05 |
|  | *238* | 0.04 |  | 0.08 |
|  | *241* | 0.07 |  | 0.16 |
|  | *244* | 0.32 |  | 0.34 |
|  | *247* | 0.15 |  | 0.09 |
|  | *250* | 0.07 |  | 0.07 |
|  | *253* | 0.03 |  | 0.02 |
|  | *256* | 0.01 |  | - |
|  | *259* | 0.01 |  | - |
